# Practices, Attitudes, and Knowledge Among Healthcare Providers and Oncologists in China Regarding Male Fertility Preservation

**DOI:** 10.3389/frph.2022.801378

**Published:** 2022-01-31

**Authors:** Chuan Huang, Ying Chun Xu, Li Hua Kuang, Qiong Yu Lan, Jing Hu, Wenbing Zhu, Liqing Fan, Qing Li

**Affiliations:** ^1^Department of Oncology, The Second Affiliated Hospital of Nanchang University, Nanchang, China; ^2^The Reproductive & Genetic Hospital of CITIC-XIANGYA, Changsha, China; ^3^Department of Oncology, Renji Hospital of Shanghai Jiaotong University, School of Medicine, Shanghai, China; ^4^Jiangxi Key Laboratory of Clinical and Translational Cancer Research, Nanchang, China; ^5^Department of Anesthesiology, South Campus, Renji Hospital, School of Medicine, Shanghai Jiao Tong University, Shanghai, China

**Keywords:** male fertility preservation, attitudes, oncologists, healthcare provider, China

## Abstract

**Objective:**

The purpose of this study was to help to promote a better understanding of the male fertility preservation status in China.

**Methods:**

In this cross-sectional survey, 1,912 healthcare providers and oncologists were surveyed anonymously using 16 questions carried out at community oncology practices in China from September 2018 to April 2021. 16 questions were designed to evaluate their knowledge on male fertility preservation in cancer patients, assess the factors they considered when deciding whether to discuss male fertility preservation with their patients.

**Results:**

Among the 1,912 healthcare providers (42.2% male), 1,713 (89.6%) considered that patients with cancer should be recommended for fertility preservation. 1,264 (66.1%) respondents were aware of male fertility preservation, but only 248 (13.0%) respondents knew the correct institutions. Whether a healthcare provide recommended fertility preservation to their patients depended on the provider's educational background, professional qualifications, hospital grade, area, department, and age. Among the healthcare providers, the three main factors for not recommending fertility preservation for patients with cancer were lack of suitability of the patient for fertility (28.2%), lack of knowledge of fertility preservation (28.6%), and lack of knowledge concerning the institutes that provide fertility preservation (25.4%).

**Conclusion:**

Despite this, healthcare providers and oncologists in China showed a positive attitude toward fertility preservation in patients with cancer. Hence, the education of physicians should include fertility preservation, with the aim of increasing their knowledge and awareness. There should be more collaboration between oncologists and reproductive medicine specialists.

## Introduction

Cancer incidence and mortality have been increasing in China, and before 45 years of age (within the reproductive window), the incidence rates of cancer in male and female patients were 193.2 and 241.7 per 100,000 individuals in 2015, respectively (1). Fertility is a vital factor among reproductive-age patients with cancer (2). Although cancer treatment often affects fertility, patients remain unaware of this possibility unless their oncologist informs them of the risk. Often, the physician is also unaware of the importance of fertility to patients unless the patients themselves bring up the topic (3). This information gap might lead to a lack of fertility preservation (FP) in patients with cancer undergoing fertility-compromising treatments.

The American Society of Clinical Oncology (ASCO) published a guideline on FP for adults and children with cancer; oncologists were recommended to discuss the possibility of infertility with reproductive-age patients and offer referrals for FP consultation (3–5). Although the patients may be initially focused on their cancer diagnosis and treatment, healthcare providers are encouraged to advise patients about the potential risk of infertility as early as possible during the treatment process to permit a wider range of FP options (3). Despite these guidelines, referrals by oncologists are made inconsistently, and many reproductive-age patients still undergo treatment without any discussion regarding FP (6).

A study in the USA determined that <50% of US physicians follow ASCO guidelines, which indicate that all patients of reproductive age should be informed about FP (7). Another survey found that most respondents agreed that a fertility consultation should be offered to all pubertal patients with cancer; however, only 46% pubertal male patients and 12% pubertal female patients with cancer referred to a fertility specialist before cancer treatment. A small-scale investigation in Lebanon found that only 69.8% of oncologists knew about the institute specializing in sperm cryopreservation for referral (8). In the Netherlands, a study found that pediatric oncologists did not possess the knowledge to sufficiently counsel these patients or refer them frequently to a fertility specialist, although they were well-aware of the effects on fertility of cancer treatment (9).

At present, there is a lack of relevant guidelines for fertility preservation in cancer patients in Mainland China. Therefore, whether clinicians recommend fertility preservation to patients in clinical practice mainly depends on the attitude of clinicians themselves. The practices, attitudes, and knowledge regarding male FP among clinical healthcare providers and oncologists in China remain unclear. In the present study, we surveyed Chinese oncologists and clinical healthcare providers to assess the status of male FP in patients with cancer in China. We hope that this interpretation will help to promote increased understanding of the status of male FP in China.

## Methods

### Study Design and Population

In the present study, a cross-sectional design was used to assess the practices, attitudes, and knowledge regarding male FP among clinical healthcare providers and oncologists and in China. The questionnaire was designed by oncologists and reproductive medicine specialists, and the revised survey was piloted with a small group of physicians to test its validity and acceptability. The survey comprised 16 questions that aimed to assess the attitudes of healthcare providers toward male FP, and the implementation of these beliefs in daily clinical practice for patients with cancer (Supplementary Figure 1). Seven questions were related to the baseline demographics of the participants. Nine questions were designed to evaluate their knowledge of male FP in patients with cancer, and to assess the factors they considered when deciding whether to discuss FP with their patients. The questionnaire was used to survey healthcare providers and oncologists working in the fields of internal medicine, pediatric medicine, oncological radiotherapy, surgery, and clinical oncology in Chinese hospitals. The participants indicated their consent to participate in the study by completing and submitting the questionnaires.

### Study Procedure

The study was performed between September 2018 and April 2021. The survey was administered via a paper questionnaire at the Chinese Society of Clinical Oncology (CSCO) and other Oncology Conferences.

The survey was explained briefly in the administered questionnaire. Subjects who agreed to participate were then prompted to complete the questionnaire. All the respondents were recruited anonymously.

### Statistical Analysis

Statistical analyses were performed using the Statistical Package for the SPSS 19.0 (IBM Corp., Armonk, NY, USA) and SAS 9.0 (SAS Institute, Cary, NC, USA). The demographic characteristics of the responders were described using means with standard deviations (SD), percentages, and frequencies. Frequencies or percentages were used to present categorical variables. To examine the association between demographic characteristics and the participants' knowledge and practice, a multivariable log-binomial regression models and chi-squared tests were used. Adjusted prevalence ratio estimates were calculated from the log-binomial regression model. Statistical significance was considered at a *p*-value of <0.05.

The Second affiliated hospital of Nanchang University Ethics Committee provided ethical approval for this study (No. 2017030).

## Results

### Sample Characteristics

A total of 3,000 questionnaires were distributed and 1980 questionnaires were collected, among which 36 questionnaires did not answer the questions completely, 32 were for scientific researchers or other staff who did not provide patient care, a total of 1,912 complete and eligible questionnaires were obtained, with a response rate of 63.73%. The demographic characteristics of the respondents are show in Table 1. The respondents were aged 22–59 years [mean age, 32 years (SD, 7.1 years)]; 807 respondents (42.2%) were male and 1,105 respondents (57.8%) were female. The respondents belonged to 29 provinces across 7 geographical regions (Northwest China, Southwest China, South China, Central China, East China, North China, and Northeast China), excluding Taiwan, Hong Kong, Macau, Tibet, and Qinghai. Among the respondents 49.9% belonged to the clinical/hematological oncology department and 81.2% s worked in Level 3-A hospitals. In addition, 34.3% of the respondents were attending physicians. With regard to educational level, 32.9% had bachelor degrees, 47.4% had master degrees, and 16.7% had doctoral degrees.

**Table 1:** Baseline characteristics of healthcare providers.

**Characteristics**		**Mean ±SD or** ***N*****(%)**
Total		1,912 (100%)
**Gender**		
Male		807 (42.2%)
Female		1,105 (57.8%)
**Age, median (range), y**		
Age distribution, y		32 ± 7.1 (22–59)
20–30		669 (34.9%)
31–40		856 (44.8%)
41–50		(16.8%)
>50		66 (3.5%)
**Province**		
Northeast		168 (8.8%)
	Heilongjiang	7 (0.3%)
	Jilin	95 (5.0%)
	Liaoning	66 (3.5%)
North China		239 (12.5%)
	Beijing	185 (9.7%)
	Tianjing	11 (0.6%)
	Shanxi	8 (0.4%)
	Hebei	20 (1.0%)
	Inner Mongoria	15 (0.8%)
East China		869 (45.4%)
	Shanghai	269 (14.1%)
	Jiangsu	73 (3.8%)
	Zhejiang	104 (5.4%)
	Anhui	37 (1.9%)
	Fujian	84 (4.4%)
	Jiangxi	273 (14.3%)
	Shandong	29 (1.5%)
Central China		285 (14.9%)
	Henan	90 (4.7%)
	Hubei	60 (3.1%)
	Hunan	135 (7.1%)
South China		245 (12.8%)
	Guangdong	153 (8.0%)
	Guangxi	75 (3.9%)
	Hainan	17 (0.9%)
Southwest		72 (3.8%)
	Sichuan	33 (1.7%)
	Guizhou	19 (1.0%)
	Yunnan	10 (0.5%)
	Chongqing	10 (0.5%)
Northwest		34 (1.8%)
	Shaanxi	13 (0.7%)
	Gansu	7 (0.4%)
	Ningxia	11 (0.6%)
	Xinjiang	3 (0.1%)
**Department**		
Clinical/hematological oncology		954 (49.9%)
Surgery		371 (19.4%)
Oncological radiotherapy		208 (10.9%)
Padiatric oncology		83 (4.3%)
Medicine		190 (9.9%)
Others		106 (5.5%)
**Grade of hospital^a^**		
Level 3-A		1,553 (81.2%)
Level 3-B		72 (3.8%)
Level 2-A		219 (11.5%)
Level 2-B		21 (1.0%)
Others		47 (2.5%)
**Professional qualifications**		
Chief/associate chief physician		396 (20.7%)
Attending physician		656 (34.3%)
Resident physician		356 (18.6%)
Residency standardized training		266 (13.9%)
Nurses		238 (12.4%)
**Education background**		
Bachelor of science in medicine		630 (32.9%)
Master		906 (47.4%)
Doctor		320 (16.7%)
Others		56 (2.9%)

a *Hospitals in China are organized according to a 3-tier system that recognizes a hospital's ability to provide medical care, medical education, and conduct medical research. Based on this, hospitals are designated as grade 1, 2 or 3. Furthermore, these three grades are further subdivided into subsidiary levels: A, B and C*.

### Knowledge, Attitudes, and Practice Regarding Male FP in Patients With Cancer

The knowledge, attitudes, and practice regarding male FP in healthcare providers are shown in Table 2. Most of the respondents (1,825; 95.4%) agreed that chemotherapy and radiotherapy may affect the fertility of patients with cancer, and a majority of respondents (1,713; 89.6%) also believed that FP should be recommend to patients with cancer. However, only 614 (32.1%) respondents actually recommended FP to their patients in daily practice. We investigated the awareness regarding FP for male patients, and found that 1,264 (66.1%) respondents were aware of male FP, 787 (41.2%) were aware of the methods, and only 248 (13%) were aware of the institutes/departments that performed male FP. Knowledge of FP was primarily obtained from the literature (45%), networks (43.7%), conferences (31.4%), media (32.5%), books (28.8%), and other avenues, such as education by senior doctors (26.6%). Among the reasons for FP recommendation to patients with cancer, the fertility needs of the patient was the most prominent (48.3%), followed by the notion that this constitutes appropriate health education for patients (38.0%), and adherence to guidelines or literature recommendations (19.7%). The three main reasons why the respondents did not recommend FP to patients with cancer included a lack of suitability of the patient for fertility (28.2%), lack of knowledge regarding FP (28.6%), and ignorance of the institutes providing FP (25.4%). All the factors that affected the recommendation decision of the respondents are shown in Table 2.

**Table 2:** Knowledge, attitudes, and practices toward male fertility preservation among oncologists and healthcare providers^a^.

	**Number (%)**
Are you aware of chemotherapy and radiotherapy threat patients' fertility?	
No	87 (4.6%)
Yes	1,825 (95.4%)
Are you aware of male fertility preservation?	
No	648 (33.9%)
Yes	1,264 (66.1%)
Do you know how to preserve male fertility?	
No	1,125 (58.8%)
Yes	787 (41.2%)
Do you know where to refer patients for male fertility preservation?	
No	1,664 (87.0%)
Yes	248 (13.0%)
Have you ever recommended patients to fertility preservation?	
No	1,298 (67.9%)
Yes	614 (32.1%)
Do you think it is necessary to recommend fertility preservation to cancer patients?	
No	199 (10.4%)
Yes	1,713 (89.6%)
How do you know about fertility preservation?	
Books	551 (28.8%)
Literature	861 (45.0%)
Conference	601 (31.4%)
Media	622 (32.5%)
Network	837 (43.7%)
Others	510 (26.6%)
Why do you recommend fertility preservation to patients?	
Provide appropriate health education for patients	727 (38.0%)
Patients have fertility needs	924 (48.3%)
Guide or literature recommendation	376 (19.7%)
Reduce medical disputes	237 (12.4%)
Others	176 (9.2%)
Why do not you recommend fertility preservation to patients?	
Patients have no fertility needs	408 (21.3%)
Lack of suitability of the patient for fertility	540 (28.2%)
Fertility preservation will delay the timing of patient treatment	392 (20.5%)
Fertility preservation will add extra psychological stress to patients	237 (12.4%)
Increase the tension between doctors and patients	120 (6.3%)
I am too busy to recommend fertility preservation to patients	86 (4.5%)
My hospital has no fertility preservation department	331 (17.3%)
I don't understand fertility preservation	546 (28.6%)
I don't know where to perform fertility preservation	485 (25.4%)
Others	136 (7.1%)

a *A total of 1,912 valid questionnaires were obtained. Frequencies and percentage were used to describe various demographic characteristics of the study participants*.

Univariate analyses found that factors such as educational background, professional qualifications, hospital grade, area, department, and age were associated significantly with the knowledge and recommendation rate for male FP (*P* < 0.01; Table 3). Male physicians had higher cognition of male fertility preservation than female physicians (*P* < 0.05), but no difference in the recommended rate of fertility preservation was observed in clinical practice (*P* = 0.203). With the increasing age, knowledge regarding male FP by physicians also increased gradually. Doctors from the oncological radiotherapy t (46.6%) and surgery departments (38%) appeared to have a higher recommendation rate. The recommended rate for male FP was as high as 40.7% in Central China, followed by Northeast China (35.7%) and South China (35.1%). In the log-binomial regression analysis, the respondents' knowledge and recommendation practice was related to department, area, Professional qualifications and education background (*P* < 0.05; Table 4).

**Table 3:** Univariate analysis of male fertility preservation cognition and practice by different characteristics of respondents [*N* (%)]^a^.

**Variable**			**Are you aware of male FP?**				**Do you know how to**				**Have you ever recommended**			
							**preserve male fertility?**				**patients to FP?**			
			**No**	**Yes**	**χ^2^**	***P***	**No**	**Yes**	**χ^2^**	***P***	**No**	**Yes**	**χ^2^**	***P***
Gender	Male	807	239 (29.6)	568 (70.4)	11.39	0.001^**^	444 (55.0)	363 (45.0)	8.41	0.032^*^	535 (66.3)	272 (33.7)	1.62	0.203
	Female	1,105	409 (37.0)	696 (63.0)			681 (61.6)	424 (38.4)			763 (69.0)	342 (31.0)		
Age	20–30	669	306 (45.7)	363 (54.3)	69.45	0.000^**^	478 (71.4)	191 (28.6)	83.19	0.000^**^	530 (79.2)	139 (20.8)	79.96	0.000^**^
	31–40	856	249 (29.1)	607 (70.9)			470 (54.9)	386 (45.1)			562 (65.7)	294 (34.3)		
	41–50	321	82 (25.5)	239 (74.5)			156 (48.6)	165 (51.4)			167 (52.0)	154 (48.0)		
	>50	66	11 (16.7)	55 (83.3)			21 (31.8)	45 (68.2)			39 (59.1)	27 (40.9)		
Department	Clinical/hematological oncology	954	313 (32.8)	641 (67.2)	58.56	0.000^**^	554 (58.1)	400 (41.9)	108.65	0.000^**^	644 (67.5)	310 (32.5)	69.23	0.000^**^
	Surgery	371	143 (38.5)	228 (61.5)			235 (63.3)	136 (36.6)			230 (62.0)	141 (38.0)		
	Oncological radiotherapy	208	33 (15.9)	175 (84.1)			79 (38.0)	129 (62.0)			111 (53.4)	97 (46.6)		
	Pediatric oncology	83	21 (25.3)	62 (74.7)			40 (48.2)	43 (51.8)			60 (72.3)	23 (27.7)		
	Medicine	190	87 (45.8)	103 (54.2)			135 (71.1)	55 (28.9)			165 (86.8)	25 (13.2)		
	Others	106	51 (48.1)	55 (51.9)			82 (77.4)	24 (22.6)			88 (83.0)	18 (17.0)		
Area	Northeast	168	60 (35.7)	108 (64.3)	33.73	0.000^**^	113 (67.3)	55 (32.7)	15.61	0.016^*^	108 (64.3)	60 (35.7)	17.48	0.008^**^
	North China	239	52 (21.8)	187 (78.2)			140 (58.6)	99 (41.4)			169 (70.7)	70 (29.3)		
	East China	869	339 (39.0)	530 (61.0)			530 (61.0)	339 (39.0)			619 (71.2)	250 (28.8)		
	Central China	285	85 (29.8)	200 (70.2)			145 (50.9)	140 (49.1)			169 (59.3)	116 (40.7)		
	South China	245	80 (32.7)	165 (67.3)			136 (55.5)	109 (44.5)			159 (64.9)	86 (35.1)		
	Southwest	72	17 (23.6)	55 (76.4)			40 (55.6)	32 (44.4)			49 (68.1)	23 (31.9)		
	Northwest	34	15 (44.1)	19 (55.9)			21 (61.8)	13 (38.2)			25 (73.5)	9 (26.5)		
Grade of hospital	Level 3-A	1,553	501 (32.2)	1,052 (67.7)	13.58	0.009^**^	887 (57.1)	666 (42.8)	19.55	0.001^**^	1,034 (66.6)	519 (33.4)	9.92	0.042^*^
	Level 3-B	72	25 (34.7)	47 (65.2)			37 (51.4)	35 (48.6)			47 (65.3)	25 (34.7)		
	Level 2-A	219	90 (41.1)	129 (58.9)			151 (68.9)	68 (31.1)			163 (74.4)	56 (25.6)		
	Level 2-B	21	12 (57.1)	9 (42.9)			17 (81.0)	4 (19.0)			17 (81.0)	4 (19.0)		
	Others	47	20 (42.6)	27 (57.4)			33 (70.2)	14 (29.8)			37 (78.7)	10 (21.3)		
Professional qualification	Chief/Associate chief physician	396	93 (23.5)	303 (76.5)	106.79	0.000^**^	179 (45.2)	217 (54.8)	97.10	0.000^**^	205 (51.8)	191 (48.2)	118.60	0.000^**^
	Attending physician	656	162 (24.7)	494 (75.3)			338 (51.5)	318 (48.5)			414 (63.1)	242 (36.9)		
	Resident physician	356	142 (39.9)	214 (60.1)			241 (67.7)	115 (32.3)			254 (71.3)	102 (28.7)		
	Residency standardized training	266	135 (50.8)	131 (49.2)			194 (72.9)	72 (27.1)			226 (85.0)	40 (15.0)		
	Nurse	238	116 (48.7)	122 (51.3)			173 (72.7)	65 (27.3)			199 (83.6)	39 (16.4)		
Education background	Bachelor	630	244 (38.7)	386 (61.3)	35.90	0.000^**^	400 (63.5)	230 (36.5)	27.40	0.001^**^	457 (72.5)	173 (27.5)	19.69	0.000^**^
	Master	906	308 (34.0)	598 (66.0)			532 (58.7)	374 (41.3)			614 (67.8)	292 (32.2)		
	Doctor	320	68 (21.3)	252 (78.8)			152 (47.5)	168 (52.5)			187 (58.4)	133 (41.6)		
	Others	56	28 (50.0)	28 (50.0)			41 (73.2)	15 (26.8)			40 (71.4)	16 (28.6)		

a *Chi-squared Test to predict the influence of demographic characteristics on participants' knowledge and practice of the three questions “are you aware of male FP,” “Do you know how to preserve male fertility,” and “Have you ever recommended patients to FP”*.

* *p < 0.05*.

** *p < 0.01*.

**Table 4:** Log-binomial regression models analysis of male fertility preservation cognition and practice by different characteristics of respondents.

	**Are you aware of male FP?**		**Do you know how to**		**Have you ever recommended**	
			**preserve male fertility?**		**patients to FP?**	
	***PR***^a^ **(95% *CI*)**	***P***-**value**	***PR***^a^ **(95% *CI*)**	***P***-**value**	***PR***^a^ **(95% *CI*)**	***P***-**value**
**Age**						
20–30	Reference	—	Reference	–	Reference	–
31–40	1.158 (1.048–1.280)	**0.004**	1.343 (1.134–1.593)	**0.001**	1.189 (0.957–1.480)	0.120
41–50	1.080(0.944–1.233)	0.260	1.255 (0.989–1.586)	0.059	1.190 (0.891–1.590)	0.239
>50	1.191 (1.007–1.237)	0.028	1.611 (1.221–1.823)	**0.001**	0.980 (0.646–1.435)	0.919
**Sex**						
Male	Reference	–	Reference	–	Reference	–
Female	0.978 (0.922–1.038)	0.466	0.985 (0.889–1.090)	0.768	1.110 (0.977–1.257)	0.112
**Department**						
Clinical/hematological oncology	Reference	–	Reference	–	Reference	–
Medicine	0.896 (0.880–0.900)	**0.004**	0.721 (0.707–0.740)	**0.001**	0.369 (0.247–0.522)	**0.000**
Oncological radiotherapy	1.080 (1.069–1.447)	**0.003**	1.245 (1.218–1.262)	**0.000**	1.266 (1.073–1.472)	**0.003**
Pediatric oncology	1.004 (0.985–1.007)	0.943	1.030 (1.013–1.046)	0.761	0.663 (0.451–0.911)	**0.021**
Surgery	0.944 (0.923–0.946)	**0.040**	0.852 (0.841–0.860)	**0.015**	1.090 (0.934–1.261)	0.261
Others	0.910 (0.902–0.926)	0.070	0.664 (0.645–0.689)	**0.004**	0.537 (0.337–0.787)	**0.004**
**Area**						
Northeast	Reference	–	Reference	–	Reference	–
North China	1.108 (1.093–1.214)	**0.031**	1.219 (0.951–1.588)	0.129	0.747 (0.572–0.979)	**0.032**
East China	0.977 (0.971–1.059)	0.611	1.243 (1.007–1.580)	0.057	0.824 (0.672–1.033)	0.075
South China	1.060 (1.045–1.576)	0.286	1.452 (1.141–1.881)	**0.003**	0.969 (0.759–1.249)	0.805
Central China	1.103 (1.094–1.361)	0.062	1.620 (1.295–2.074)	**0.000**	1.250 (0.999–1.589)	0.058
Northwest	0.889 (0.853–0.982)	0.304	1.125 (0.670–1.691)	0.612	0.664 (0.341–1.097)	0.163
Southwest	1.139 (1.129–1.143)	0.059	1.403 (1.002–1.918)	**0.039**	0.878 (0.587–1.249)	0.496
**Grade of hospital**						
Level 3-A	Reference	–	Reference	–	Reference	–
Level 3-B	0.951 (0.788–1.096)	0.545	1.018 (0.785–1.251)	0.882	0.984 (0.691–1.310)	0.921
Level 2-A	0.875 (0.775–0.973)	**0.021**	0.740 (0.596–0.899)	**0.004**	0.788 (0.612–0.989)	0.051
Level 2-B	0.638 (0.349–0.951)	0.074	0.456 (0.152–0.930)	0.081	0.616 (0.205–1.258)	0.282
Others	0.858 (0.647–1.054)	0.215	0.706 (0.428–1.033)	0.119	0.671 (0.357–1.073)	0.150
**Professional qualifications**						
Chief and associate chief physician	Reference	–	Reference	–	Reference	–
Attending physician	1.056 (0.962–1.160)	0.264	1.073 (0.905–1.273)	0.421	1.055 (0.962–1.160)	0.264
Resident physician	0.854 (0.732–0.991)	**0.042**	0.755 (0.575–0.981)	**0.040**	0.854 (0.732–0.991)	**0.042**
Residency standardized training	0.701 (0.579–0.845)	**0.000**	0.644 (0.462–0.888)	**0.008**	0.701 (0.579–0.845)	**0.000**
Nurse	0.734 (0.618–0.864)	**0.000**	0.648 (0.482–0.857)	**0.003**	0.734 (0.618–0.864)	**0.000**
**Education background**						
Bachelor of science in medicine	Reference	–	Reference	–	Reference	–
Master	1.169 (0.962–1.123)	0.345	1.043 (0.919–1.188)	0.524	1.040 (0.890–1.223)	0.624
Doctor	1.099 (1.010–1.199)	**0.030**	1.112 (0.959–1.291)	0.159	1.104 (0.917–1.330)	0.294
Others	0.961 (0.716–1.188)	0.757	1.049 (0.639–1.528)	0.830	1.640 (1.059–2.035)	**0.008**

*PR, prevalence ratio; 95% CI, 95% confidence intervals*.

a *The main effect of only one independent variable on the dependent variable was analyzed in each model, and the other independent variables were set as covariables and included in the analysis. The bold values mean p < 0.05*.

## Discussion

The fertility problems of patients with cancer primarily arise from the use of chemotherapy and radiotherapy. These treatments cause dysfunction of the gonadal glands, and can affect reproductive health (4). Male spermatogonia are particularly sensitive to radiation and chemotherapy (10). Thus, cancer treatments might lead to infertility. The European Society for Medical Oncology (ESMO) and the ASCO recommend that as standard strategies for male FP, sperm should be cryopreserved (4, 5). The National Comprehensive Cancer Network (NCCN) Adolescent and Young Adult (AYA) Oncology guidelines also suggested that the oncologists should talk to their patients about the risk to fertility and make referrals if necessary to a center specializing in FP (www.NCCN.org). Given the importance of FP, other countries such as Germany (11), Japan (12), and South Korea (13) have specific guidelines for FP in patients with cancer. However, in China, no comprehensive guidelines regarding FP in patients with cancer are currently established. In recent years, patients have become more concerned about their current reproductive health status and the potential for conceiving after cancer treatment (14). Moreover, since the one-child policy in China was terminated in 2015, the fertility needs of patients have increased. Hence, according to the patient's fertility needs, FP should be performed as early as possible before cancer treatment. However, we know little about the practices and attitudes of oncologists regarding FP of their patients.

As far as we know, the present study was the first large-scale survey to examine the practices attitudes, and knowledge among oncologists and healthcare providers in China regarding of FP in patients with cancer. The level of knowledge among oncologists with regard to FP was unsatisfactory, only 66.1% of oncologists reported that they were aware of male FP. In addition, the NCCN guidelines specify that cryopreservation of sperm for male patients with cancer is a standard FP method. However, fewer than 40% of physicians could describe these methods. Furthermore, as far as specific sites of FP were concerned, 87% of respondents admitted that they were unaware of the institutes specializing in male FP. Only 32.1% of respondents declared that they had recommended a patient with cancer for FP in daily clinical practice. In the USA, 98% of oncology physicians said they usually discussed the issue of future fertility with their patients and 97% had referred patients with fertility questions to a specialist (15).

This result suggests inadequate discussions regarding male FP in China and the lack of knowledge concerning FP would indicate a training gap. Many participants in this survey had never received any FP-related training, and 45% of participants only knew about FP through the literature. However, we are encouraged by the foundation of the China Society for Fertility Preservation (CSFP), which held its first congress on FP in Hainan in 2017. This represents a major platform to improve FP-related training and the promotion of information exchange among Chinese oncology professionals. Meanwhile, the Chinese Medical Doctor Association also published the first Chinese male fertility preservation consensus.

The present study identified that individuals currently undergoing standardized residency training, were less aware of FP. The reasons might be the lack of education regarding FP in their training, their short period of clinical experience, and a tendency to follow the recommendation of a senior physician rather than actively communicate with the patient about FP. Therefore, we recommend that in China, further fertility-related education should be developed and delivered to oncologists. In addition, we also recommend that the importance of FP should be emphasized and standardized in medical education. It is worth mentioning that oncological radiotherapy physicians had the highest awareness of FP, followed by clinical/hematological oncology and pediatric oncologists. However, general medical practitioners, including those involved in the fields of gastroenterology, respiratory medicine, nephrology, and cardiovascular medicine, had the lowest awareness, and also had the lowest recommendation rate. Therefore, although these general medical practitioners encounter patients with cancer in clinical practice, their awareness of FP for such patients was weak. Therefore, further training programs for fertility-related issues should developed and provided to other departments, not just oncologists, in China.

We also found that there were significant differences in the recommendation rate and awareness of FP among physicians in different areas of China. These differences could be related to the importance of sperm banks to male FP in different areas. In China, sperm banks are the only institutions for male FP. On April 11, 2017, the National Health and Family Planning Commission of China issued the “List of Medical Institutions Approved for Human Sperm Banks.” According to the list, Mainland China has 27 human sperm banks. Oncologists in North, South, and Central China had a higher recommendation rate and awareness of FP. The Human Sperm Bank of the National Research Institute for Family Planning in Beijing, China (CNHSB), is in Northern China and has been offering male FP for more than 10 years. However, from July 2006 to December 2017, only 145 male patients with cancer patients underwent sperm cryopreservation, only 9.7% (14 out of 145) of patients returned to use their cryopreserved sperm for assisted reproduction technology (ART), and the rate of patients who had a baby was 71.4% (10 out of 14) as of June 2018 (16). The Human Sperm Bank of Reproductive & Genetic Hospital of CITIC-Xiangya in Hunan, which is in Central China, was the first Hunan human sperm bank to open in China (1981). Although oncologists in Central China had highest rate of recommendation of FP, only 97 male patients with cancer underwent sperm cryopreservation from February 2004 to March 2015 (17). The Guangdong Human Sperm Bank is in South China, in which 288 male patients with cancer underwent sperm cryopreservation from June 2003 to June 2016 (18). Therefore, in China, communication between Human Sperm banks and oncologists needs to improve.

In the present survey, we assessed the difficulties encountered by healthcare providers and patients with cancer with regard to FP issues. The main reason for the non-recommendation of FP to patients by respondents is the perception that the patient is unsuitable for fatherhood (28.39%). Nevertheless, research suggests that the children born to cancer survivors have no significantly increased risk of developing congenital anomalies resulting the mutagenic cancer treatment applied to their parent (19). A systematic review reports the sperm cryopreservation and reproductive outcome in male cancer patients, the aggregated rate of use of cryopreserved semen was 8%, the rate of patients who used their frozen semen and achieved parenthood 49% (20). So “cancer patient is not suitable for fertility” is misleading, cancer patients have a good chance of maintain their reproductive potential and lead a normal and fulfilled family life with a healthy child. “I am not aware of FP” and "I do not know where FP is performed” were the other two major reasons for the non-recommendation of FP. Moreover, like most oncologists, 20.69% of the respondents were concerned that FP would delay timely cancer treatment. However, sperm cryopreservation is an effective method of FP for male patients, which does not delay timely cancer treatment. Hence, a lack of understanding of FP by oncologists is a major reason for the lack of provision of timely and effective FP recommendations in patients with cancer. In the future, knowledge regarding male FP should be enhanced among oncologists by providing systematic training programs. In addition, we should consider setting up an insurance system to cover the cost of FP technologies, and consumer laws related to FP should be revised if necessary. However, these oncologists had a positive attitude despite their lack of knowledge of FP, 89.6% of respondents believed that patients with cancer should be recommended to receive FP. Therefore, more FP training in China will be required in the future.

There are some limitations associated with the present study, including the unbalanced sample size. The survey population comprised a higher ratio of clinicians working in Level 3-A hospitals, which could have resulted in self-selection bias (i.e., an increased willingness to participate in the study). Moreover, we may have been more likely to receive a response from clinicians who are interested in this area. In some provinces, the sample size was relatively small, which might have resulted in unrepresentative findings. Nevertheless, this was the first study to evaluate the practices, attitudes, and knowledge related to FP in clinicians in China. This study provides important information that can be applied to improve overall knowledge of FP, and could be used to produce educational materials and design training courses for clinicians.

## Conclusions

In conclusion, Chinese oncologists and healthcare providers have a positive attitude regarding FP for patients with cancer. However, they lack knowledge regarding FP techniques and the institutes specializing in these procedures. Hence, FP should be included in the medical education curriculum to increase knowledge and awareness among young physicians. Furthermore, there are no comprehensive guidelines on FP for various cancers in China; therefore, oncologists and reproductive medicine specialists should work collaboratively to develop such guidelines for Chinese patients with cancer and should attempt to improve the awareness of FP guidelines among healthcare providers. Additional conferences on FP in patients with cancer should be held, and reproductive medicine specialists should be invited to offer special lectures at such conferences, thus enhancing the communication between the two disciplines. Meanwhile, there should be more communication between human sperm bank providers and oncologists. The findings of the present study reveal the similarities and differences between Western countries and China in terms of patients' reproductive rights and may be applicable to other Asian countries.

## Data Availability Statement

The original contributions presented in the study are included in the article/Supplementary Material, further inquiries can be directed to the corresponding author/s.

## Ethics Statement

The studies involving human participants were reviewed and approved by the Second Affiliated Hospital of Nanchang University Ethics Committee (Ethics approval number: 2017030). The patients/participants provided their written informed consent to participate in this study.

## Author Contributions

Material preparation, data collection, and analysis were performed by CH, YX, LK, QL, JH, WZ, LF, and QL. The first draft of the manuscript was written by CH and QL. All authors commented on previous versions of the manuscript, read, approved the final manuscript, and contributed to the study conception and design.

## Funding

This study was funded by grants from Jiangxi youth science fund project in China (grant number: 20181BAB215029).

## Conflict of Interest

The authors declare that the research was conducted in the absence of any commercial or financial relationships that could be construed as a potential conflict of interest.

## Publisher's Note

All claims expressed in this article are solely those of the authors and do not necessarily represent those of their affiliated organizations, or those of the publisher, the editors and the reviewers. Any product that may be evaluated in this article, or claim that may be made by its manufacturer, is not guaranteed or endorsed by the publisher.

## References

[1] Chen W Zheng R Baade PD Zhang S Zeng H Bray F. Cancer statistics in China, 2015. CA Cancer J Clin. (2016) 66:115–32. 10.3322/caac.21338 26808342

[2] Maltaris T Seufert R Fischl F Schaffrath M Pollow K Koelbl H. The effect of cancer treatment on female fertility and strategies for preserving fertility. Eur J Obstet Gynecol Reprod Biol. (2007) 130:148–55. 10.1016/j.ejogrb.2006.08.006 16979280

[3] Loren AW Mangu PB Beck LN Brennan L Magdalinski AJ Partridge AH. Fertility preservation for patients with cancer: American Society of Clinical Oncology clinical practice guideline update. J Clin Oncol. (2013) 31:2500–10. 10.1200/JCO.2013.49.2678 23715580 PMC5321083

[4] Lee SJ Schover LR Partridge AH Patrizio P Wallace WH Hagerty K. American Society of Clinical Oncology recommendations on fertility preservation in cancer patients. J Clin Oncol. (2006) 24: 2917–31. 10.1200/JCO.2006.06.5888 16651642

[5] Oktay K Harvey BE Partridge AH Quinn GP Reinecke J Taylor HS. Fertility preservation in patients with cancer: ASCO clinical practice guideline update. J Clin Oncol. (2018) 36:1994–2001. 10.1200/JCO.2018.78.1914 29620997

[6] Shnorhavorian M Harlan LC Smith AW Keegan TH Lynch CF Prasad PK. Fertility preservation knowledge, counseling, and actions among adolescent and young adult patients with cancer: a population-based study. Cancer. (2015) 121:3499–506. 10.1002/cncr.29328 26214755 PMC4734641

[7] Quinn GP Vadaparampil ST Lee JH Jacobsen PB Bepler G Lancaster J. Physician referral for fertility preservation in oncology patients: a national study of practice behaviors. J Clin Oncol. (2009) 27:5952–7. 10.1200/JCO.2009.23.0250 19826115

[8] Ghazeeri G Zebian D Nassar AH Harajly S Abdallah A Hakimian S. Knowledge, attitudes and awareness regarding fertility preservation among oncologists and clinical practitioners in Lebanon. Hum Fertil. (2016) 19:127–33. 10.1080/14647273.2016.1193636 27376977

[9] Overbeek A van den Berg M Louwe L Wendel E ter Kuile M Kaspers G. Practice, attitude and knowledge of Dutch paediatric oncologists regarding female fertility. Neth J Med. (2014) 72:264–70. 24930460

[10] Moss JL Choi AW Fitzgerald Keeter MK Brannigan ER. Male adolescent fertility preservation. Fertil Steril. (2016) 105:267–73. 10.1016/j.fertnstert.2015.12.002 26707904

[11] von Wolff M Montag M Dittrich R Denschlag D Nawroth F Lawrenz B. Fertility preservation in women–a practical guide to preservation techniques and therapeutic strategies in breast cancer, Hodgkin's lymphoma and borderline ovarian tumours by the fertility preservation network FertiPROTEKT. Arch Gynecol Obstet. (2011) 284:427–35. 10.1007/s00404-011-1874-1 21431846 PMC3133651

[12] Suzuki N. Clinical practice guidelines for fertility preservation in pediatric, adolescent, and young adults with cancer. Int J Clin Oncol. (2019) 24:20–7. 10.1007/s10147-018-1269-4 29582194

[13] Kim J Kim SK Hwang KJ Kim HS. Fertility Fertility preservation during cancer treatment: The Korean Society for Fertility Preservation clinical guidelines. Clin Exp Reprod Med. (2017) 44:171–74. 10.5653/cerm.2017.44.4.171 29376012 PMC5783912

[14] Flink DM Kondapalli LA Kellar-Guenther Y. Priorities in fertility decisions for reproductive-aged cancer patients: fertility attitudes and cancer treatment study. J Adolesc Young Adult Oncol. (2017) 6:435–43. 10.1089/jayao.2016.0072 28221816 PMC5649415

[15] Rosenberg SM Gelber S Gelber RD Krop E Korde LA Pagani O. Oncology physicians' perspectives on practices and barriers to fertility preservation and the feasibility of a prospective study of pregnancy after breast cancer. J Adolesc Young Adult Oncol. (2017) 6:429–34. 10.1089/jayao.2017.0031 28686476 PMC5649410

[16] Fu L Zhou F An Q Zhang K Wang X Xu J. Sperm cryopreservation for male cancer patients: more than 10 years of experience, in Beijing China. Med Sci Monit. (2019) 25:3256–61. 10.12659/MSM.913513 31048670 PMC6511111

[17] liu X Zhu W Fan L. Analysis on the characteristics of 149 male reproductive insurance population. Zhonghua Nan Ke Xue Za Zhi. (2015) 21:1146–7. 10.13263/j.cnki.nja.2015.12.0199782046

[18] Ma C Zhuang J Deng S Ma T Tang Y Luo L. Retrospective analysis of reproductive insurance and clinical application of human sperm bank in Guangdong Province for 13 years. Guangdong Med J. (2017) 38:748–50. 10.13820/j.cnki.gdyx.2017.05.017

[19] Signorello LB Mulvihill JJ Green DM Munro HM Stovall M Weathers RE. Congenital anomalies in the children of cancer survivors: a report from the childhood cancer survivor study. J Clin Oncol. (2012) 30:239–45. 10.1200/JCO.2011.37.2938 22162566 PMC3269950

[20] Ferrari S Paffoni A Filippi F Busnelli A Vegetti W Somigliana E. Sperm cryopreservation and reproductive outcome in male cancer patients: a systematic review. Reprod Biomed Online. (2016) 33:29–38. 10.1016/j.rbmo.2016.04.002 27156003

