# Supplementary material for: Practices, Attitudes, and Knowledge Among Healthcare Providers and Oncologists in China Regarding Male Fertility Preservation
Source: Front Reprod Health. 2022 Jan 31;4:801378. doi: 10.3389/frph.2022.801378 (PMC9580752; doi:10.3389/frph.2022.801378)
Supplement: Supplementary file 1 [file Data_Sheet_1.PDF]

Physician/nurse attitudes and perceptions of fertility preservation in cancer patients

Gender：☐ male ☐ female	Age：	Province：

Department: ☐ Clinical/haematological oncology ☐ Surgery ☐ Oncological radiotherapy ☐ Paediatric oncology ☐ Medicine ☐ Others

Grade of hospital： ☐ Level 3-A ☐ Level 3-B ☐ Level 2-A ☐ Level 2-B ☐ Others：

Professional qualifications： ☐ Chief/Associate chief physician ☐ Attending physician ☐ Resident physician ☐ Residency standardized training ☐ Nurses

Education background： ☐ Bachelor of Science in medicine ☐ Master ☐ Doctor ☐ Others

Are you aware of chemotherapy and radiotherapy threat patients' fertility? ☐ No ☐ Yes

Are you aware of male fertility preservation? ☐ No ☐ Yes

Do you know how to preserve male fertility? ☐ No ☐ Yes

Do you know where to refer patients for male fertility preservation?

☐ Yes（If you know, please write down the organizations you know）____________ ☐ No

How do you know about fertility preservation? ☐ Books ☐ Literatures ☐ Conference ☐ Media ☐ Network ☐ Others：

Have you ever recommended patients to fertility preservation? ☐ No ☐ Yes

Why did you choose yes to the above question? (multiple choices) (Why do you recommend fertility preservation to patients?)	☐ Provide appropriate health education for patients<br>☐ Patients have fertility needs<br>☐ Guide or literature recommendation<br>☐ Reduce medical disputes<br>☐ Others： ________ (If you have a different opinion, please write here)
Why did you choose No to the above question? (multiple choices) (Why do not you recommend fertility preservation to patients?)	☐ Patients have no fertility needs<br>☐ Lack of suitability of the patient for fertility<br>☐ Fertility preservation will delay the timing of patient treatment<br>☐ Add extra psychological stress to patients<br>☐ Increase the tension between doctors and patients<br>☐ I worked too busy to recommend fertility preservation to patients<br>☐ My hospital has no fertility preservation department<br>☐ I don’t understand fertility preservation<br>☐ I don’t know where to perform fertility preservation<br>☐ Others： ________ (If you have a different opinion, please write here)

Do you think it is necessary to recommend fertility preservation to cancer patients? ☐ No ☐ Yes
